# Dynamic Changes in Macrophage Activation and Proliferation during the Development and Resolution of Intestinal Inflammation

**DOI:** 10.4049/jimmunol.1400502

**Published:** 2014-09-26

**Authors:** Matthew C. Little, Rebecca J. M. Hurst, Kathryn J. Else

**Affiliations:** Faculty of Life Sciences, University of Manchester, Manchester M13 9PT, United Kingdom

## Abstract

Macrophages (Mφs) accumulate at sites of inflammation, and, because they can assume several functionally distinct states of activation, they can either drive or restrain inflammatory responses. Once believed to depend on the recruitment of blood monocytes, it is now clear that the accumulation of Mφs in some tissues can result from the proliferation of resident Mφs in situ. However, little is known about the proliferation and activation state of Mφ subsets in the gut during the development and resolution of intestinal inflammation. We show that inflammatory Mφs accumulate in the large intestine of mice during the local inflammatory response to infection with the gastrointestinal nematode parasite *Trichuris muris*. Classically activated Mφs predominate initially (as the inflammation develops) and then, following worm expulsion (as the inflammation resolves), both the resident and inflammatory populations of Mφs become alternatively activated. A small but significant increase in the proliferation of inflammatory Mφs is seen but only during the resolution phase of the inflammatory response following both worm expulsion and the peak in Mφ accumulation. In contrast to recent studies in the pleural and peritoneal cavities, the proliferation of resident and alternatively activated Mφs does not increase during the inflammatory response. Furthermore, in CCR2^−/−^ mice, monocyte recruitment to the gut is impeded, and the accumulation of alternatively activated Mφs is greatly reduced. In conclusion, the recruitment of blood monocytes is the principle mechanism of Mφ accumulation in the large intestine. This study provides a novel insight into the phenotype and behavior of intestinal Mφ during infection-driven inflammation.

## Introduction

Macrophages (Mφs) are mononuclear phagocytes of the innate immune system and are involved in host-defense, metabolism, and the homeostatic regulation of healthy tissues. Playing diverse and contrasting roles, Mφs can initiate, amplify, and regulate the adaptive immune system and both drive and resolve inflammatory responses. The gut is the largest reservoir of Mφs in the body (1), and intestinal Mφs play a key role in driving the pathogenesis of inflammatory bowel disease (2).

Mφs can assume several functionally different states of activation that are regulated by the prevailing cytokine milieu and other factors that are present at sites of inflammation. Mφs respond to IFN-γ, with or without LPS, to become classically activated (3, 4). Classically activated Mφs (M1s) play a vital role in Th1-mediated immunity against intracellular pathogens and are characterized by the expression of inducible NO synthetase (iNOS) (3, 4). In contrast, IL-4 and IL-13 induce the alternative activation of Mφs by signaling through IL-4Rα (4), the common subunit of their receptors. Associated with both Th2-mediated allergic reactions and responses to a range of phylogenetically distinct helminth parasites (5), alternatively activated Mφs (M2s) are characterized by their expression of arginase-1, resistin-like molecule α (RELMα), and Ym-1 (4).

Distinct resident and inflammatory subpopulations of Mφs exist in tissues, including the gut. Much of our understanding of the functional specialization of Mφ subsets has been through the development of CX3CR1^gfp/+^ transgenic mice, which express eGFP under the control of the CX3CR1 promoter (6). CX3CR1^hi^ resident Mφs and CX3CR1^int^ inflammatory Mφs can be easily identified by their differential expression of eGFP (7). Resident Mφs in the gut are involved in homeostasis and the prevention of inflammatory reactions against commensal bacteria and food proteins (8). In most tissues (including the brain, liver, spleen, and lungs), resident Mφs are derived during embryogenesis from cells in the yolk sac and fetal liver, and after birth, they are maintained by self-renewal (9–11). However, the origin of gut-resident Mφs appears to be unique because they are derived from Ly6C^hi^CX3CR1^lo^ blood monocytes (12–14).

During the development of colitis, inflammatory Mφs accumulate in the inflamed mucosa, where they produce TNF-α and other proinflammatory mediators (14–17). They are recruited from Ly6C^hi^CCR2^hi^CX3CR1^lo^ blood monocytes in a CCR2-dependent mechanism and drive the inflammatory response (14, 15, 17). However, during an inflammatory response in the pleural and peritoneal cavities, resident Mφs proliferate. Therefore, in these tissues, Mφ accumulation during inflammation can be accomplished independent of monocyte recruitment (18–20). However, in the gut, it remains to be determined whether the proliferation of Mφs acts in tandem with the recruitment of blood monocytes to promote the accumulation of Mφs during the development and resolution of inflammation.

*Trichuris muris*, a natural nematode parasite of mice that resides in the cecum and proximal colon, is a model for the human whipworm *Trichuris trichiura*, which infects as many as one billion people worldwide (21). Resistance to a high-level infection with *T. muris* varies considerably between different strains of mouse. Many strains, such as BALB/c, mount a protective Th2 response to *T. muris*, leading to the rapid expulsion of the parasite, whereas others, such as C57BL/6, mount a mixed Th1/Th2 response and expel the parasite more slowly. In contrast, susceptible strains, such as AKR, mount an inappropriate Th1 response and fail to expel *T. muris* (22, 23). Furthermore, a low-level infection also induces a Th1 response, and this confers susceptibility to all strains of mouse (24). Importantly, regardless of the underlying adaptive immune response, the large intestine becomes inflamed as Mφs, and other leukocytes, accumulate in the tissue (23).

By exploiting this natural model of intestinal inflammation, we describe the dynamic changes that take place to Mφ subtypes and their activation states as inflammation develops and resolves. Furthermore, we use CX3CR1^gfp/+^ transgenic mice and multiparameter flow cytometry to distinguish among resident, inflammatory, and M2 subsets of Mφs and assess their proliferation in the intestine.

## Materials and Methods

### Mice

Specific pathogen-free AKR, BALB/c, and C57BL/6 mice were purchased from Harlan. CX3CR1^gfp/+^ mice were bred at the University of Manchester. CCR2^−/−^ mice were purchased from The Jackson Laboratory. All strains of mouse were maintained in individually ventilated cages. Only the males were used in experiments when they were 6–12 wk old. The mouse studies were reviewed and approved by the Home Office and performed under the strict legal requirements of the Animal (Scientific Procedures) Act 1986 (as amended).

### Parasite

The E strain of *T. muris* was maintained as described previously (25). *T. muris* excretory/secretory (E/S) Ags were prepared by culturing adult worms in vitro at 37°C for 4 h (25). *T. muris* eggs were administered, by oral gavage, resulting in either a low-level infection (35 eggs given) or a high-level infection (200 eggs given).

### Cell culture

Mesenteric lymph node (MLN) cells were cultured and stimulated with 50 μg/ml *T. muris* E/S Ags for 48 h as previously described (23). The culture supernatants were harvested and stored at −20°C until they were assayed for cytokines.

### Multiplex quantification of cytokines

A Cytometric Bead Array kit (BD Biosciences, Oxford, U.K.) was used in accordance with the manufacturer’s instructions to assay cytokines using an LSR II flow cytometer (BD Biosciences).

### Isolation of lamina propria leukocytes

Lamina propria leukocytes (LPLs) were isolated from the proximal colon and cecum by enzymatic digestion as previously described (14, 15).

### Proliferation

Two approaches were taken to measure proliferation. Firstly, mice were injected i.p. with BrdU, which is incorporated into the newly synthesized DNA of replicating cells during the S phase of the cell cycle. The mice were killed 4 h later, and an Ab was used to detect the BrdU in the DNA of Mφs by flow cytometry (as described next). Secondly, an Ab was used to measure Ki-67 in Mφs by flow cytometry. This nuclear protein regulates cell division and is present during all active phases of the cell cycle (G_1_, S, G_2_, and M) but is absent from quiescent cells (G_0_).

### Flow cytometry

The LPLs were washed in Flow Cytometry Buffer (PBS containing Ca^2+^ and Mg^2+^, with 4% FCS and 0.05% w/v sodium azide) and then incubated with rat, anti-mouse CD16/32 mAb (eBioscience, Hatfield, U.K.) for 30 min on ice to block FcR. The cells were then stained with the following Abs to extracellular markers for 30 min on ice: PE rat, anti-mouse F4/80 mAb (eBioscience), Alexa Fluor 700 hamster, anti-mouse CD11c mAb (eBioscience), allophycocyanin–eFluor 780 rat, anti-mouse CD11b mAb (eBioscience), PerCP-Cy5.5 rat, anti-mouse F4/80 mAb (eBioscience), biotin rat, anti-mouse MHC class II (I-A/I-E) mAb (eBioscience) used in conjunction with PE-Vio770 mouse, anti-biotin mAb (Miltenyi Biotec, Bisley, U.K.), and VioGreen rat, anti-mouse CD45 mAb; or PE rat, anti-mouse CD103 mAb (BD Biosciences), PE rat, anti-mouse Siglec-F mAb (BD Biosciences), PE rat, anti-mouse Ly6G (BD Biosciences), FITC rat, anti-mouse CD11b mAb (eBioscience), PerCP-Cy5.5 rat, anti-mouse F4/80 mAb (eBioscience), Alexa Fluor 700 rat, and anti-mouse CD45 mAb (eBioscience). To detect live and dead cells, a Live/Dead Fixable Dead Cell Stain Kit (the Blue Fluorescent Reactive Dye version) was used according to the manufacturer’s instructions (Life Technologies). An allophycocyanin BrdU Flow kit was then used according to the manufacturer’s instructions to detect BrdU that had been incorporated into the cells (BD Biosciences). As recommended, the staining of intracellular proteins was performed at the same time using the following Abs: eFluor450 rat, anti-mouse Ki-67 mAb (eBioscience) and rabbit, anti-mouse RELMα polyclonal Ab (PeproTech, London, U.K.) used in conjunction with Qdot 605 donkey, anti-rabbit IgG (Life Technologies). To precisely control the gating for the staining of Ki-67, BrdU, and RELMα, the following control Abs were used in parallel for each mouse: rat IgG2a eFluor 450 isotype control (eBioscience), rat IgG_1_ allophycocyanin isotype control (eBioscience), and rabbit IgG control (PeproTech; used in conjunction with Qdot 605 donkey, anti-rabbit IgG), respectively.

### Immunohistochemistry

Immunohistochemistry was performed on frozen cross-sections of proximal colon using standard immunoperoxidase techniques as described previously (23). The following primary Abs were used: biotin rat, anti-mouse CD4 mAb (5 μg/ml; BD Biosciences), biotin rat, anti-mouse CD45 mAb (2 μg/ml; BD Biosciences), biotin rat, anti-mouse F4/80 mAb (2 μg/ml; AbD Serotec, Oxford, U.K.), rabbit, anti-mouse RELMα polyclonal Ab (2 μg/ml; PeproTech), goat, anti-mouse Arginase-1 polyclonal Ab (1 μg/ml; Santa Cruz Biotechnology, from Insight Biotechnology, Wembley, U.K.), goat, anti-mouse Ym-1 (Chitinase 3-like 3/ECF-L) polyclonal Ab (2 μg/ml; R&D Systems, Abingdon, U.K.), or rabbit, anti-mouse iNOS polyclonal Ab (1 μg/ml; Santa Cruz Biotechnology). The following secondary Abs were then used: for Arginase-1 and Ym-1 staining, we used biotin rabbit, anti-goat IgG F(ab′)_2_ (1/2000 v/v; Millipore, Watford, U.K.), and for iNOS and RELMα, we used biotin goat, anti-rabbit IgG F(ab′)_2_ (1/600 v/v; Santa Cruz Biotechnology). The appropriate isotype control mAbs or polyclonal control IgGs were used in parallel sections. The color development was monitored and was stopped, by washing in PBS, before any false-positive staining occurred in the isotype control sections. The sections were counterstained in Haematoxylin QS (Vector Laboratories). After randomization and blinding of the slides, the number of positively stained cells was determined in each section by light microscopy. The staining was performed in triplicate, and all of the positively stained cells in each section were counted (as a guide, there are ∼200 crypts in each section).

### Statistics

Statistical analysis was performed by the Kruskal–Wallis test with Dunn’s posttest (using GraphPad Prism software; GraphPad).

## Results

### Following infection with *T. muris*, Mφs accumulate in the large intestine of C57BL/6 mice, where they are the predominant type of infiltrating leukocyte

**FIGURE 1.**
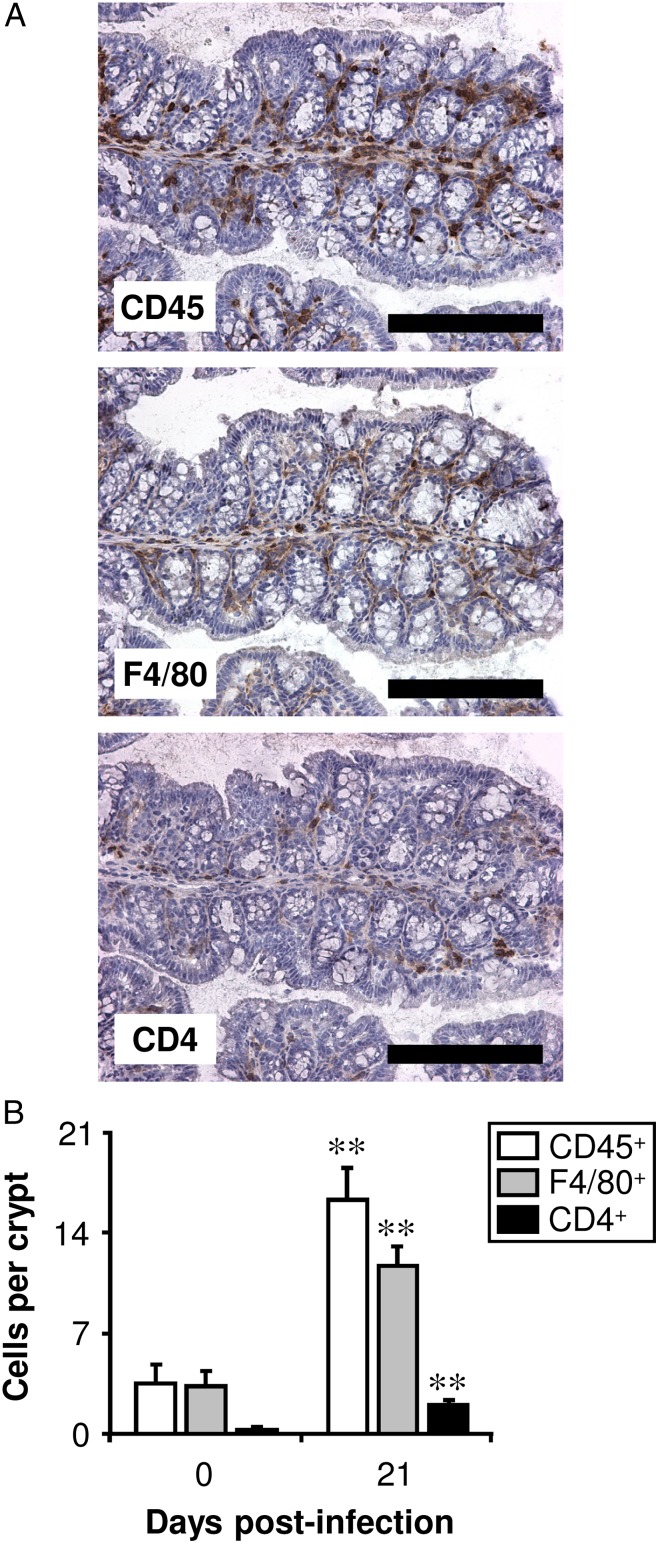
Mφs are the predominant type of leukocyte in the large intestine both before and postinfection with *T. muris*. C57BL/6 mice were either left uninfected or infected with a high level of *T. muris* ova. Immunohistochemical staining of leukocytes (CD45^+^), Mφs (F4/80^+^), or Th cells (CD4^+^) was conducted on sections of the proximal colon. (**A**) Representative photographs are shown of serial sections from one mouse, 21 d postinfection. Scale bars, 200 μm. Quantitative analysis of the staining in both uninfected (0 d postinfection) and infected (21 d postinfection) mice is shown in (**B**). The values represent the means + SEM of between five and seven mice in each group, and the results are representative of three separate experiments. **p < 0.01 (21 d postinfection compared with uninfected).

The detection of CD45, F4/80, and CD4 by immunohistochemistry allowed the number of leukocytes, Mφs, and Th cells, respectively, to be quantified in the proximal colon of C57BL/6 mice. In uninfected mice, >90% of the leukocytes were Mφs (Fig. 1). Following a high-level infection with *T. muris*, leukocytes accumulated in the large intestine. There was a significant increase in the number of both Mφs and Th cells in the proximal colon 21 d postinfection, and ∼80% of the leukocytes were Mφs (Fig. 1). Similar values were found in a previous study in BALB/c and AKR strains of mouse (23). Eosinophils (analyzed by the immunohistochemical staining of Siglec-F) also accumulated in the large intestine postinfection (in uninfected mice, there were 0.2 ± 0.1 Siglec-F^+^ cells per crypt compared with 2.8 ± 1.7 cells/crypt 21 d postinfection, data not shown). Eosinophils are known to express F4/80 as well as Mφs. However, because Siglec-F^+^ cells were much less abundant than F4/80^+^ cells, only a small fraction of the F4/80^+^ cells were potentially eosinophils.

### Following a high-level infection, the adaptive immune response and the ability to expel *T. muris* are strain dependent

**FIGURE 2.**
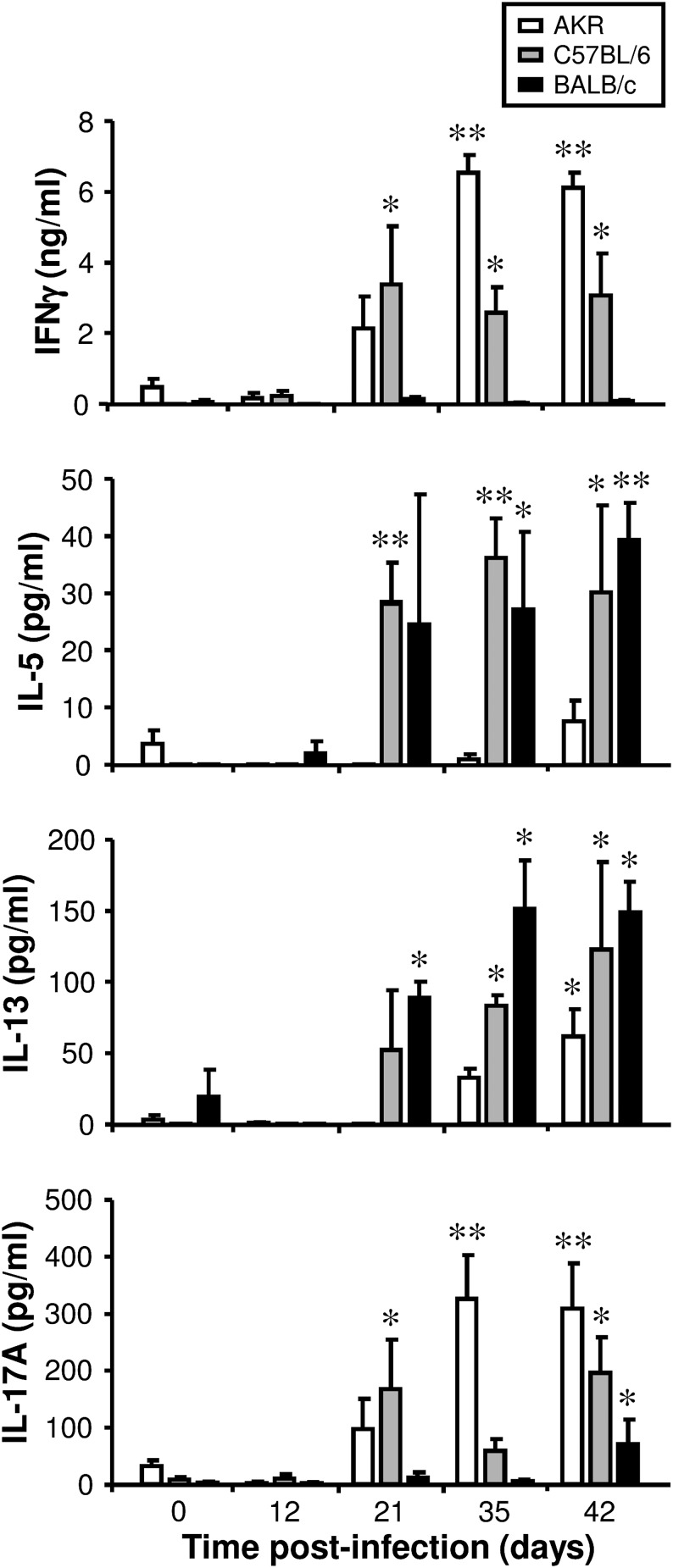
The adaptive immune response following *T. muris* infection. Three different strains of mouse (AKR, C57BL/6, and BALB/c) were either left uninfected or infected with a high level of *T. muris* ova. MLN cells were isolated from uninfected mice (0 d postinfection) and infected mice at various time points postinfection and stimulated in vitro for 48 h with *T. muris* E/S Ag. The supernatant was analyzed for cytokines using a Cytokine Bead Array kit. The values are the means + SEM of five mice in each group. The experiment was repeated at days 0 and 21 only. *p < 0.05, **p < 0.01 (time points postinfection compared with uninfected).

**FIGURE 3.**
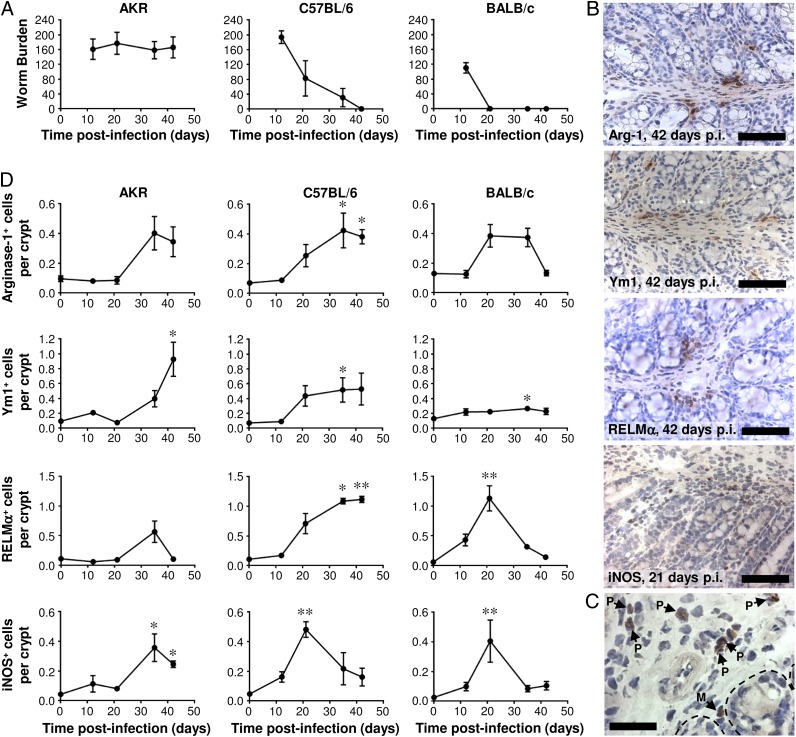
The temporal relationship between *T. muris* expulsion and the accumulation of M1s and M2s in the large intestine. Three different strains of mouse (AKR, C57BL/6 and BALB/c) were either left uninfected or infected with a high level of *T. muris* ova, and the number of worms in the cecum was determined at various time points postinfection (**A**). Immunohistochemical staining of M2s (either arginase-1^+^, Ym1^+^, or RELMα^+^ cells) or M1s (iNOS^+^ cells) was conducted on sections of the proximal colon. Representative photographs of the staining are shown for infected C57BL/6 mice in (**B**) at the indicated time points postinfection. Scale bars, 75 μm. An example of iNOS^+^ polymorphonuclear (P) and mononuclear (M) leukocytes 21 d postinfection in BALB/c mice is shown in (**C**). Scale bar, 30 μm. The bases of the epithelial crypts are indicated by dotted lines. Quantitative analysis of the mononuclear cell staining is shown in (**D**). The values are the means ± SEM of five mice in each group and are representative of two separate experiments. *p < 0.05, **p < 0.01 (time points postinfection compared with uninfected).

After day 35 postinfection, Ag-stimulated MLN cells from AKR mice released high levels of IFN-γ and IL-17A. Furthermore, on day 42, IL-13, but not IL-5, was also released (Fig. 2), revealing that AKR mice mounted strong Th1 and Th17 responses (and also a delayed and muted Th2 response) to the parasite. In contrast, Ag-stimulated MLN cells from BALB/c mice produced high levels of IL-5 and IL-13, but not IFN-γ postinfection. This was accompanied by a small but significant increase in IL-17A on day 42 (Fig. 2). Therefore, BALB/c mice mounted a strong Th2 response (and also a weak and delayed Th17 response) to *T. muris*. MLN cells from C57BL/6 mice released high levels of all four cytokines after day 21 postinfection (Fig. 2). Therefore, C57BL/6 mice mounted strong Th1, Th2, and Th17 responses. AKR mice failed to expel *T. muris* and a chronic infection ensued. In contrast, BALB/c and C57BL/6 mice were both resistant. However, BALB/c mice expelled the parasite more rapidly than C57BL/6 mice (Fig. 3A).

### The emergence of M1s and M2s in the large intestine postinfection follows a distinct pattern in each strain of mouse, reflecting the kinetics of worm expulsion and/or the underlying adaptive immune response

Immunohistochemical staining for the M1 marker iNOS and the M2 markers Arginase-1, Ym1, and RELMα, allowed these cells to be quantified in the proximal colon. In all three strains of mouse, there was a significant increase in the number of iNOS^+^ mononuclear leukocytes (henceforth referred to as M1s). In BALB/c and C57BL/6 mice, the number of M1s reached a peak 21 d postinfection and then subsequently decreased. In contrast, in AKR mice, the M1s emerged later and they persisted (Fig. 3D). In each of the three strains of mouse, there was a trend toward an increase in the number of Arginase-1^+^, Ym-1^+^, and RELMα^+^ mononuclear leukocytes postinfection. However, in AKR mice, the only significant increase was for Ym-1^+^ cells (Fig. 3D), and therefore, it is uncertain whether M2s emerged in this strain of mouse. In BALB/c and C57BL/6 mice postinfection, the accumulation of M2s in the large intestine (based on all three M2 markers) was clearer, and it reached a peak following worm expulsion (Fig. 3A, 3D). Surprisingly, Ym1^+^ cells were the least abundant in the most Th2-biased strain of mouse, namely BALB/c, reinforcing the need to analyze multiple markers to define alternative activation. Interestingly, in C57BL/6 mice, M1s emerged in the gut during worm expulsion, whereas M2s were most abundant following worm expulsion after the number of M1 had diminished. Both before and postinfection, the M1s and M2s were mainly situated in the lamina propria (Fig. 3B) and smooth muscle (not shown) compartments of the gut: they were rarely encountered in the intraepithelial niche of the mucosa (Fig. 3B). After day 21 postinfection, some iNOS^+^ and RELMα^+^ (but not Arginase-1^+^ or Ym-1^+^) eosinophil-like polymorphonuclear leukocytes were also observed (Fig. 3C). However, these cells were not quantified.

### The analysis of LPLs by flow cytometry confirms the emergence of M2s in the large intestine postinfection

**FIGURE 4.**
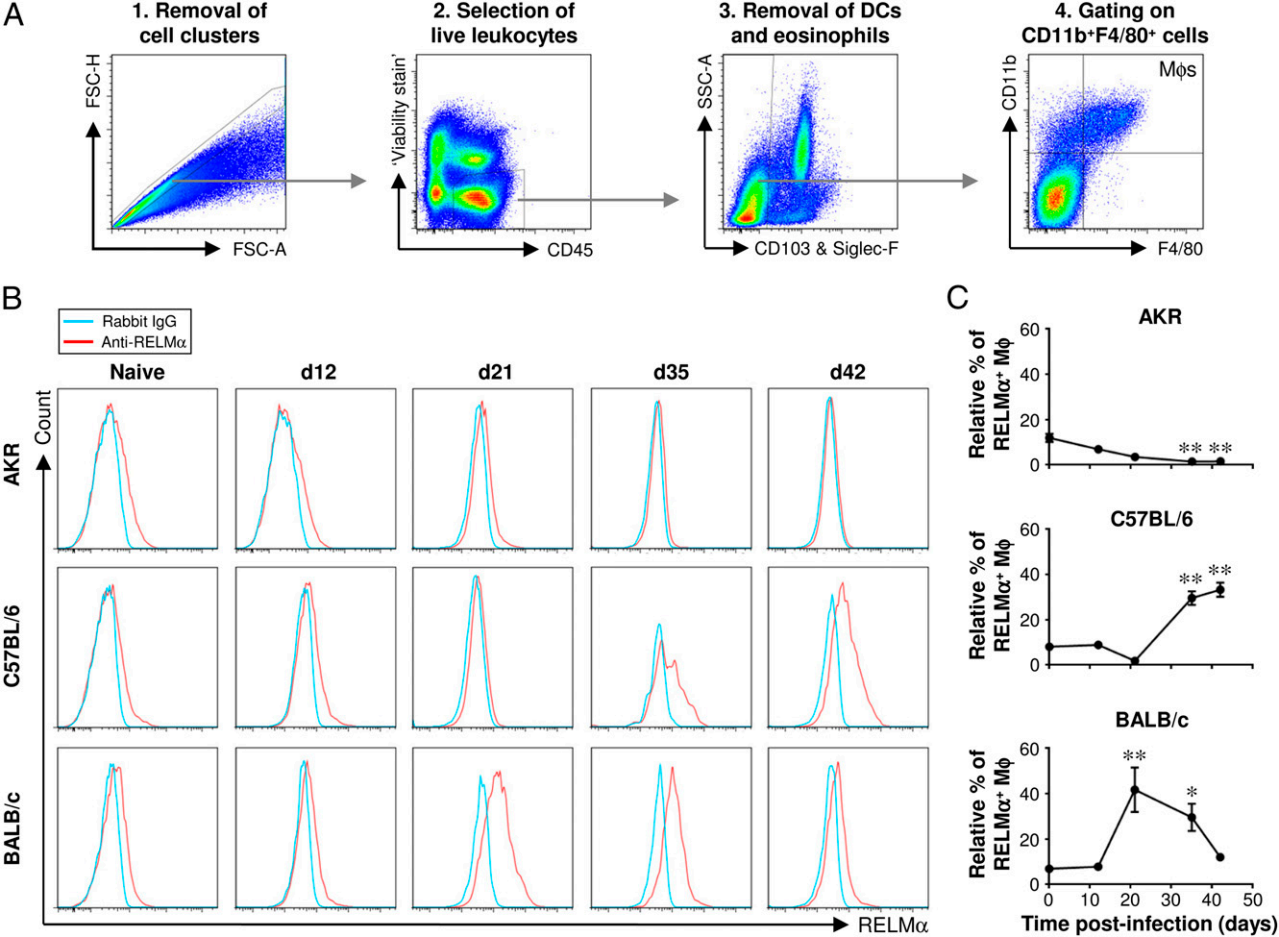
Flow cytometric analysis of lamina propria Mφs confirms the kinetics of M2 accumulation in the large intestine postinfection. Three different strains of mouse (AKR, C57BL/6, and BALB/c) were either left uninfected or infected with a high level of *T. muris* ova. Cells were isolated from the lamina propria of the cecum and proximal colon, stained with a panel of fluorochrome-labeled Abs, and then analyzed by flow cytometry. Live Mφs were analyzed by gating on viability stain–negative CD45^+^CD11b^+^F4/80^+^CD103^−^Siglec-F^−^ cells as shown in (**A**). Representative histogram plots of RELMα staining are shown in (**B**). Quantitative analysis of the staining is shown in (**C**). The values are the means ± SEM of five mice in each group, and the results are representative of two separate experiments. *p < 0.05, **p < 0.01 (time points postinfection compared with uninfected).

LPLs were liberated from the lamina propria and stained with a panel of fluorochrome-labeled Abs. A series of gating steps was performed to exclude cell clusters and doublets, select live leukocytes, and exclude eosinophils and dendritic cells (DCs) from the subsequent analysis. The F4/80^+^CD11b^+^ cells were defined as Mφs and selected for downstream analysis (Fig. 4A). Paradoxically, although the number of leukocytes in the large intestine increases postinfection with *T. muris* (Fig. 1), infected gut tissue yields fewer leukocytes from the lamina propria than uninfected tissue. As reported previously (23), the immunopathological disruption to the gut postinfection appears to interfere with the isolation of leukocytes from the lamina propria leading to an artificially low yield. Therefore, because it cannot be determined reliably postinfection, the flow cytometry data were expressed not as total numbers of M2s but instead as the relative percentage of M2s within the total Mφ population.

The marker RELMα was chosen for the analysis of M2s by flow cytometry because it exhibited similar staining profiles to Arginase-1 and Ym1, yet it revealed the greatest differences between uninfected and infected mice (Fig. 3D). In all three strains of mouse, ∼10% of Mφs from the lamina propria of the large intestine, in its resting state, were alternatively activated. In AKR mice, the relative percentage of M2s decreased gradually postinfection (Fig. 4B, 4C). Conversely, in both C57BL/6 and BALB/c mice, the relative percentage of M2s increased postinfection showing that about one-third of the Mφs were alternatively activated. Reaching a peak after worm expulsion, the accumulation of M2s reflected the different kinetics of worm expulsion between these two strains of mouse (Fig. 4B, 4C), recapitulating the earlier observations made by immunohistochemistry (Fig. 3).

A minor fraction of CD103^+^ DCs also expressed RELMα in all three strains of mouse, and there was a small but significant increase in the relative percentage of these RELMα^+^ DCs 42 d postinfection in C57BL/6 and BALB/c mice (Supplemental Fig. 1A–C). Approximately 5% of eosinophils also expressed RELMα, but there was no significant change postinfection (Supplemental Fig. 2).

### Five contrasting subpopulations of CX3CR1^+^ myeloid cells can be defined in the lamina propria of the large intestine

**FIGURE 5.**
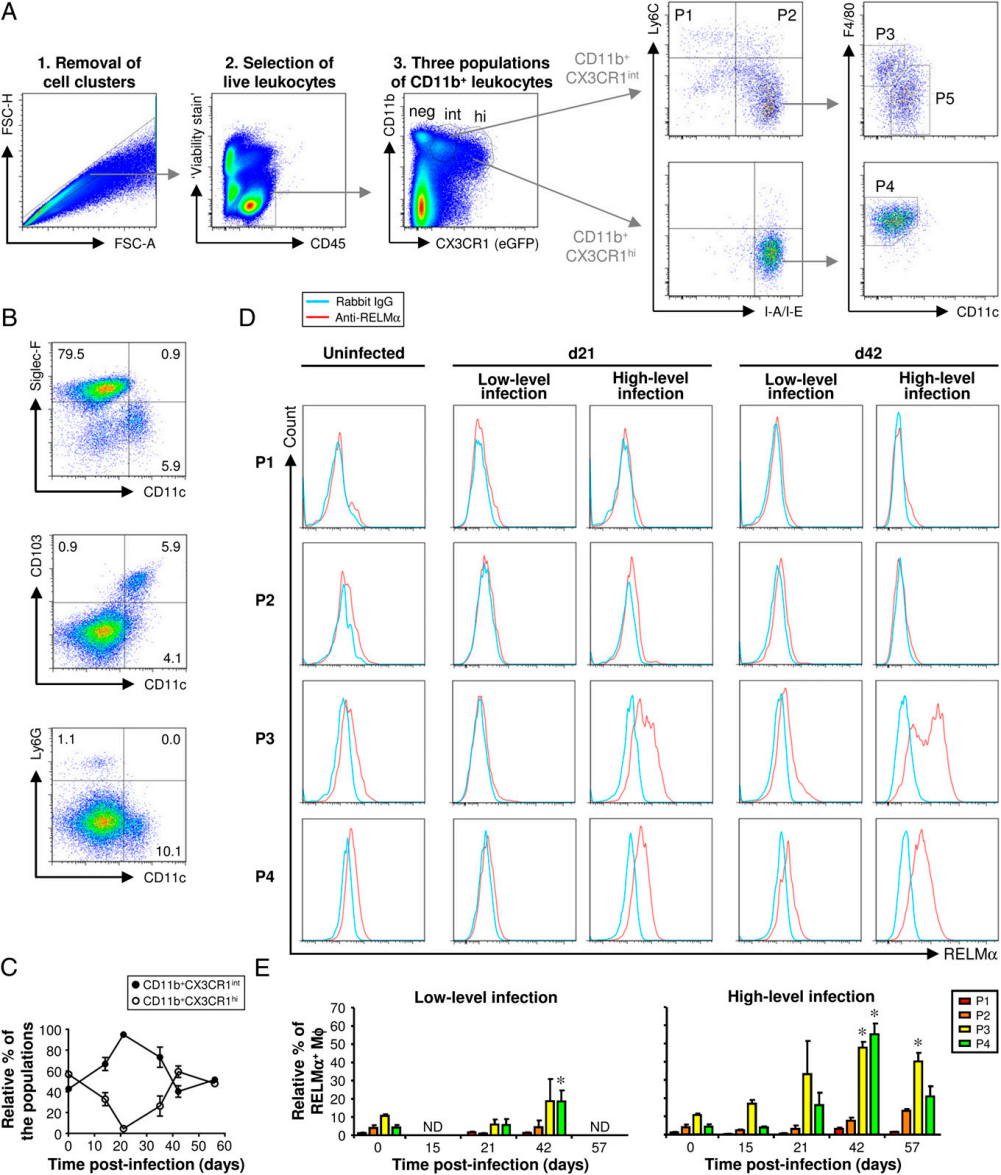
In the large intestine of CX3CR1^gfp/+^ mice, five populations of myeloid cells can be defined (P1–P5). M2s emerge postinfection in populations P3 and P4 (both of which are subpopulations of Mφs). Following a high-level infection, the accumulation of M2s in the large intestine reaches a peak after the worms have been expelled. In contrast, following a low-level infection (where the worms are not expelled), the accumulation of M2s is less marked. CX3CR1^gfp/+^ mice were infected with either a low or high level of *T. muris* ova. Another group of CX3CR1^gfp/+^ mice was left uninfected. Cells were isolated from the lamina propria of the cecum and proximal colon, stained with a panel of fluorochrome-labeled Abs, and then analyzed by flow cytometry. Live leukocytes were analyzed by gating on viability stain–negative CD45^+^ cells (**A**). Three populations of CD11b^+^ leukocytes were identified by their differential expression of eGFP (CX3CR1) (A). The CD11b^+^CX3CR1^−^ cells were analyzed, and representative plots are shown in (**B**). The relative abundance of the CD11b^+^CX3CR1^int^ and CD11b^+^CX3CR1^hi^ populations over the time course of a high-level infection is shown in (**C**). CD11b^+^CX3CR1^+^ cells could be subdivided into five populations (P1 to P5) based on their differential expression of CX3CR1 and the presence or absence of Ly6C, I-A/I-E, F4/80, and CD11c. Representative plots illustrate how these different populations of cells were defined (A). Representative histogram plots of RELMα staining in populations P1 to P4 are shown for uninfected mice and for infected mice at selected time points (**D**). The data are shown at all time points in (**E**), where the values are the means + SEM of five mice in each group, and the results are representative of two separate experiments. *p < 0.05 (time points postinfection compared with uninfected). FSC-A, forward light scatter area; FSC-H, forward light scatter height; ND, not done.

In CX3CR1^gfp/+^ mice (on a C57BL/6 background), three distinct populations of CD11b^+^ leukocytes were identified by their differential expression of eGFP (Fig. 5A). Firstly, there was a population of CD11b^+^CX3CR1^−^ leukocytes (Fig. 5A) comprised mainly of Siglec-F^+^ eosinophils and smaller populations of CD11c^+^CD103^+^ DCs and Ly6G^+^ neutrophils (Fig. 5B) (14). Secondly, there was a population of CD11b^+^ leukocytes expressing high levels of CX3CR1 (Fig. 5A), which was more prevalent in the large intestine of uninfected mice (Fig. 5C). The vast majority of these CX3CR1^high^ leukocytes were Ly6C^−^IA/IE^+^F4/80^+^CD11c^−^ (subpopulation P4 in Fig. 5A), matching the phenotype of resident Mφs as reported previously by others (14, 16). Thirdly, a population of CD11b^+^ leukocytes expressing intermediate levels of CX3CR1 (Fig. 5A) was prevalent postinfection with *T. muris* (Fig. 5C) and could be subdivided into four subpopulations (P1, P2, P3, and P5) as follows. The first subpopulation (P1) expressed Ly6C but not IA/IE and was therefore consistent with inflammatory monocytes (14, 26) (Fig. 5A). The second, a relatively small subpopulation (P2), was Ly6C^+^IA/IE^+^. Based on previous phenotypic and functional analysis (14), these cells were thought to be immature inflammatory Mφs derived from recently recruited inflammatory monocytes. The Ly6C^−^IA/IE^+^ leukocytes were heterogeneous (Fig. 5A), consisting of an F4/80^+^CD11c^−^ subpopulation [P3, thought to be mature inflammatory Mφs (14)] and an F4/80^−^CD11c^+^ subpopulation [P5, thought to be DCs (14)].

### Alternative activation occurs specifically in the resident and mature inflammatory Mφ subpopulations

Using the careful gating strategy described above (Fig. 5A), we went on to investigate which subpopulations of Mφs became alternatively activated in response to *T. muris* infection in CX3CR1^gfp/+^ mice (on a C57BL/6 background). The relative percentage of monocytes (P1), immature inflammatory Mφs (P2), mature inflammatory Mφs (P3), and resident Mφs (P4) expressing RELMα was analyzed. Furthermore, to establish whether a Th2 response and worm expulsion was required for the accumulation of M2 in the large intestine, two disparate strategies of *T. muris* infection were employed: firstly, the familiar high-level infection protocol that resulted in a mixed Th1/Th2 response and worm expulsion [with the same kinetics that was observed for wild-type (WT) C57BL/6 mice (Fig. 3A), not shown]; and secondly, a low-level infection protocol that, contrastingly, resulted in a Th1 response and chronic infection (not shown).

Hardly any monocytes (P1) expressed the M2 marker RELMα (Fig. 5D, 5E). In uninfected mice, only a small proportion of Mφs (subpopulations P2–P4) were alternatively activated (Fig. 5D, 5E). However, following a high-level infection, M2s emerged, and they were observed in the mature inflammatory (P3) and mature resident (P4) Mφ subpopulations (Fig. 5D, 5E). After worm expulsion, approximately half of the Mφs within these subpopulations were alternatively activated (Fig. 5E). As late as day 57 postinfection, a significant proportion of the mature inflammatory Mφ subpopulation (P3) was alternatively activated (Fig. 5E). M2s also emerged following a low-level (chronic) infection, but this was restricted to the mature resident Mφ subpopulation (P4) and was less marked when compared with a high-level (acute) infection (Fig. 5D, 5E). Therefore, the highest level of M2 accumulation was observed following worm expulsion.

A minor fraction of CX3CR1^+^ DCs (P5) expressed RELMα, but there was no significant difference postinfection (Supplemental Fig. 1D–F).

### A small but significant increase in the proliferation of mature inflammatory Mφs occurs in the large intestine following worm expulsion

**FIGURE 6.**
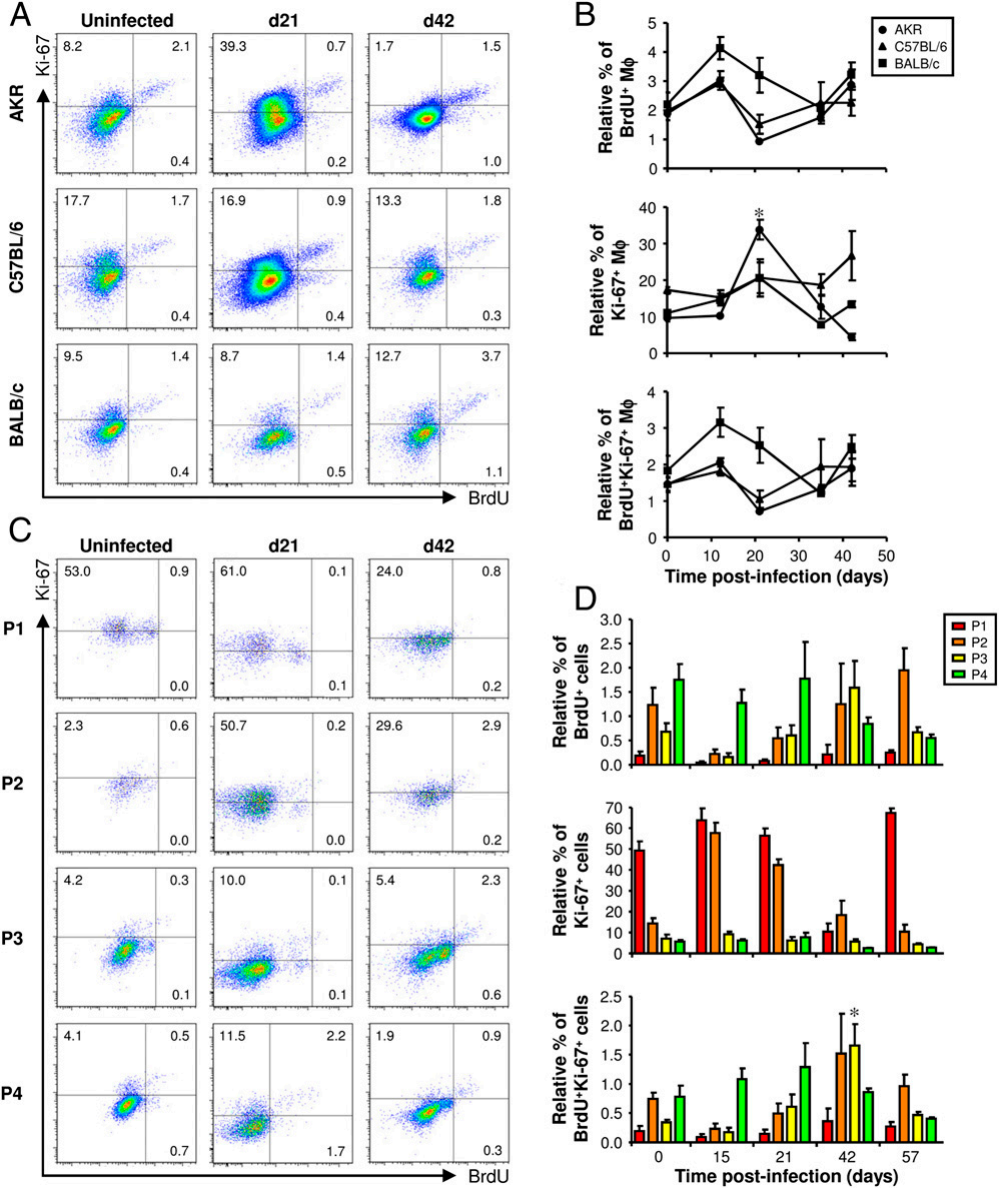
The proliferation of Mφs following infection with *T. muris*. AKR, C57BL/6, BALB/c, and CX3CR1^gfp/+^ mice were infected with a high level of *T. muris* ova. Each mouse was injected with 1.5 mg BrdU 4 h before it was killed. Cells were isolated from the lamina propria of the cecum and proximal colon, stained with a panel of fluorochrome-labeled Abs, and then analyzed by flow cytometry. In AKR, C57BL/6, and BALB/c mice, live Mφs were analyzed by gating on viability stain–negative CD45^+^CD11b^+^F4/80^+^CD103^−^Siglec-F^−^ cells (as shown in Fig. 4A). Representative plots of Ki-67 and BrdU staining are shown at selected time points postinfection (**A**). The data are shown at all time points in (**B**), where the values are the means ± SEM of five mice in each group, and the results are representative of two separate experiments. Ki-67 and BrdU staining in CX3CR1^gfp/+^ mice was analyzed by gating on each of the four populations of monocytes and Mϕs (P1–P4, as defined in Fig. 5A). Representative plots at selected time points postinfection are shown in (**C**). The gates were defined by staining with fluorochrome-labeled isotype control Abs in parallel (shown in Supplemental Fig. 3). The data are shown at all time points in (**D**) where the values are the means + SEM of five mice in each group, and the results are representative of two separate experiments. *p < 0.05 (time points postinfection compared with uninfected).

In uninfected mice, ∼2% of Mφs in the large intestine had incorporated BrdU into their DNA. As expected, most of the BrdU^+^ Mφs also expressed Ki-67 (Fig. 6A, 6B, Supplemental Fig. 3A). Therefore, a small number of Mφs proliferated in the large intestine in its resting state. Postinfection no significant increase in the relative percentage of BrdU^+^ or BrdU^+^Ki-67^+^ Mφs was detected (Fig. 6A, 6B), suggesting that proliferation does not account for the accumulation of Mφs following infection with *T. muris* in any of the different strains of mouse. Approximately 10 times more Mφs were Ki-67^+^ than BrdU^+^ reflecting the broader scope of Ki-67 as a marker of proliferation than BrdU. At 21 d postinfection, there was a significant increase in the relative percentage of Ki-67^+^ Mφs but only in AKR mice. However, this is difficult to interpret as a bona fide increase in the proliferation of Mφs because it was not accompanied by an increase in the number of BrdU^+^ cells (Fig. 6A, 6B).

**FIGURE 7.**
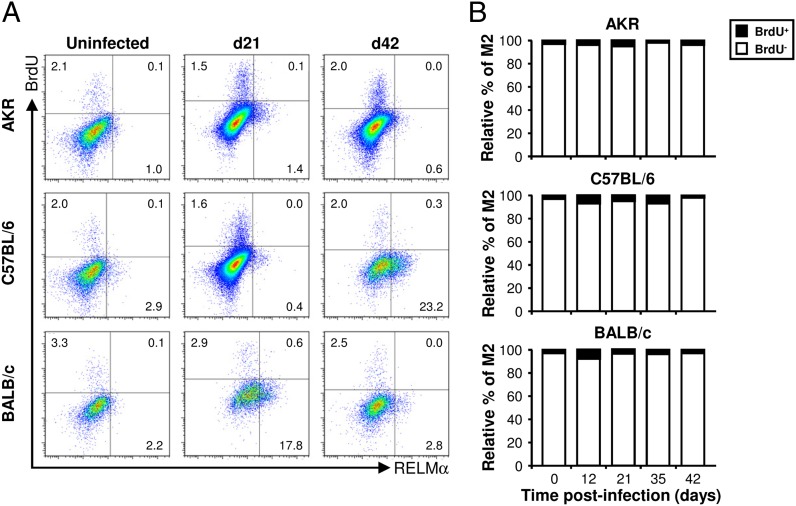
The vast majority of M2s do not proliferate. Three different strains of mouse (AKR, C57BL/6, and BALB/c) were infected with a high level of *T. muris* ova. Each mouse was injected with 1.5 mg BrdU 4 h before it was killed. Cells were isolated from the lamina propria of the cecum and proximal colon, stained with a panel of fluorochrome-labeled Abs, and then analyzed by flow cytometry. Live Mφs were analyzed by gating on viability stain–negative CD45^+^CD11b^+^F4/80^+^CD103^−^Siglec-F^−^ cells (as shown in Fig. 4A). Representative histogram plots of RELMα and BrdU staining are shown at selected time points postinfection (**A**). The RELMα^+^ cells (M2s) were then analyzed for their BrdU content: the data are shown as the relative percentage of the BrdU^+^ and BrdU^−^ populations at all time points postinfection (**B**). The values are the means of five mice in each group, and the results are representative of two separate experiments.

Despite not observing a significant increase in the percentage of BrdU^+^ Mφs postinfection, we investigated whether a small increase could have been overlooked because it was restricted to one of the subpopulations of Mφs. Interestingly, after the worms had been expelled, there was a small but significant increase in the percentage of BrdU^+^Ki-67^+^ mature inflammatory Mφs (P3), suggesting that proliferation may contribute to the accumulation of this subpopulation of Mφs in the large intestine postinfection (Fig. 6C, 6D, Supplemental Fig. 3B). However, in contrast to peritoneal and pleural cavity (18, 20), the vast majority of BrdU^+^ Mφs in the colon were RELMα^−^ (Fig. 7), implying that few of the M2s proliferated. Therefore, the accumulation of M2s in the large intestine postinfection is probably not driven by their proliferation in situ.

### The accumulation of Mφs and M2s in the large intestine postinfection is greatly reduced in CCR2-deficient mice

**FIGURE 8.**
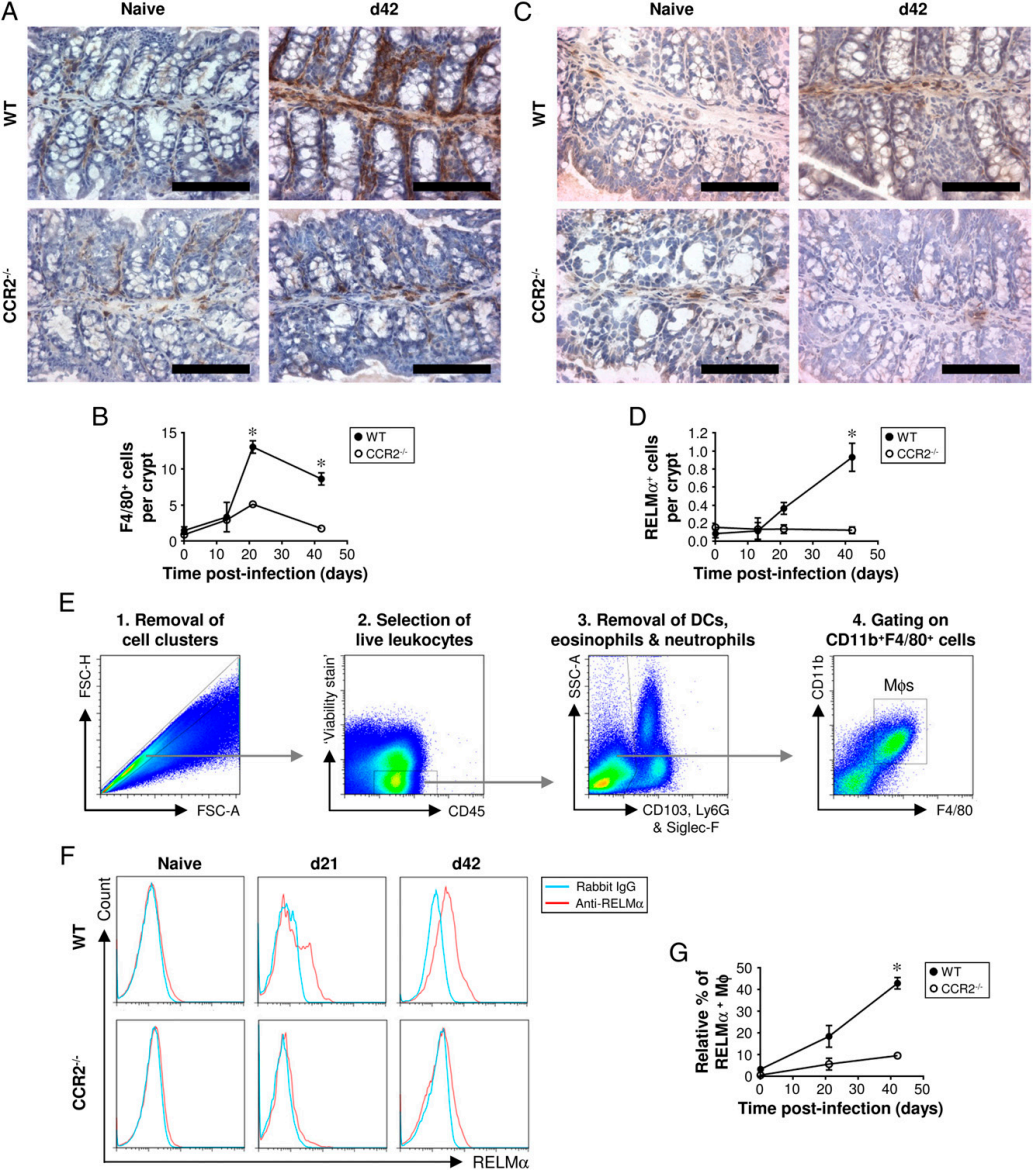
In mice lacking CCR2, the accumulation of Mφs and M2s in the colon postinfection is greatly reduced. CCR2^−/−^ and WT control mice (C57BL/6) were either left uninfected or infected with a high level of *T. muris* ova. Immunohistochemical staining of Mφs (F4/80^+^ cells) was conducted on sections of the proximal colon. Representative photographs of the F4/80 staining are shown in (**A**), and the quantitative analysis is shown in (**B**). Immunohistochemical staining of M2s (RELMα^+^ cells) was also performed on sections of the proximal colon. Representative photographs of the RELMα staining are shown in (**C**), and the quantitative analysis is shown in (**D**). Scale bars, 100 μm. Cells were isolated from the lamina propria of the cecum and proximal colon, stained with a panel of fluorochrome-labeled Abs, and then analyzed by flow cytometry. Live Mφs were analyzed by gating on viability stain–negative CD45^+^CD11b^+^F4/80^+^CD103^−^Ly6G^−^Siglec-F^−^ cells (as shown in **E**). Representative plots of RELMα staining are shown in (**F**), and the data are shown graphically in (**G**). The values are the means ± SEM of five mice in each group. *p < 0.05 (CCR2^−/−^ compared with WT at the same time point).

It has been shown previously that the recruitment of blood monocytes to the intestine is CCR2 dependent (14, 15, 17). Therefore, we used CCR2^−/−^ mice to inhibit monocyte chemotaxis to investigate whether blood-derived monocytes give rise to the Mφs and M2s that accumulate in the large intestine postinfection. CCR2^−/−^ mice (on a C57BL/6 background) were resistant to a high-level infection with *T. muris* (not shown) as reported previously (27). Immunohistochemical staining for F4/80 revealed that, in the absence of infection, Mφs resided in the lamina propria of the colon in both CCR2^−/−^ mice and their WT controls (Fig. 8A, 8B). In WT mice, Mφs accumulated in the colon, reaching a peak 21 d postinfection (Fig. 8B). In contrast, there was no significant accumulation of Mφs in CCR2-deficient mice (Fig. 8B). Low numbers of RELMα^+^ M2s were detected in the uninfected gut of both CCR2^−/−^ and WT mice by immunohistochemistry (Fig. 8C, 8D). In WT mice, the number of M2s increased postinfection, reaching a peak at day 42 (Fig. 8D). However, in CCR2^−/−^ mice, there was no increase in the number of M2s in the colon postinfection (Fig. 8D). The significant accumulation of M2s in the large intestine of WT mice, but not CCR^−/−^ mice, was confirmed by flow cytometry (Fig. 8E–G). Therefore, to a large extent, the accumulation of M2s in the intestine postinfection is driven by the CCR2-dependent recruitment of monocytes from the blood.

## Discussion

Our basic understanding of Mφ physiology has been revolutionized by the recent discovery that tissue-resident Mφs can proliferate in situ. In some tissues, this acts not only as a mechanism for the maintenance of resident Mφ numbers (9–11) but also enables the accumulation of Mφs at sites of inflammation independent of monocyte recruitment from the blood (18–20). However, whether this translates to all inflamed tissues remains to be determined. This study describes the activation state and proliferation of resident and inflammatory Mφs in the large intestine during both acute and chronic inflammation driven by the nematode parasite *T. muris*.

In resistant strains of mouse, the expulsion of *T. muris* precedes the accumulation of M2s, and the peak accumulation of M2s is reached after worm expulsion. In BALB/c mice, the gradual reduction in the number of M2s following worm expulsion probably reflects the return of the gut to a steady state after the loss of the parasites. M2s have been shown to play pivotal role in the expulsion of the gastrointestinal nematode *Heligomosoides polygyrus bakeri* during Th2 memory responses to a secondary infection (28). However, a role for M2s in the expulsion of the nematode *Nippostrongylus brasiliensis* is controversial (29, 30). In a previous study, we showed that disrupting the function of M2s (by inhibiting their arginase-1 activity) has no effect on the expulsion of *T. muris* (31). Accordingly, in this study we show that mice are resistant to *T. muris* even when the accumulation of M2s to the large intestine is inhibited. Therefore, M2s are not required for the expulsion of *T. muris*. Instead, because of the kinetics of M2 accumulation, our data support a role for M2s in the gut following worm expulsion, during the resolution phase of the inflammatory response. This is consistent with the ability of M2s to restrain the potentially damaging immunopathology following infection with nematode parasites (29, 32, 33) and a role for M2s in tissue repair and remodeling (34, 35).

Interestingly, in C57BL/6 mice, the wavelike accumulation of M1s is observed in the gut, reaching a peak around the time of worm expulsion and then receding as M2s begin to accumulate (during worm expulsion) and then predominate (after worm expulsion). Indeed, a similar transition from M1s to M2s has been observed following infections with parasites as diverse as *Taenia crassiceps*, *Schistosoma mansoni*, and *Trypanosoma congolense* (36, 37), and it is possible that the dynamics of M1 and M2 accumulation reflects sequential changes in the local cytokine milieu. However, the factors that drive this switch remain to be determined. It is possible to reprogram polarized Mφs in vitro, so that M1s can be transformed into M2s and vice versa, by switching the cytokine stimulus (38). This remarkable plasticity of Mφs may also occur in vivo because M2s seem to convert to M1s in artherosclerotic lesions (38). However, it still remains unclear whether the switch from M1 to M2 represents the recruitment of naive Mφ precursors or involves the re-education of the same Mφs in situ.

Using published approaches to define monocyte and Mφ subsets by multiparameter flow cytometry (14, 16, 26), we demonstrate, for the first time to our knowledge, the dynamic changes that occur to resident and inflammatory gastrointestinal Mφs during an inflammatory response to infection. We confirm that CX3CR1^high^ resident Mφs are the predominant population in the uninfected large intestine, although CX3CR1^int^ inflammatory Mφs are also encountered (14, 15). Postinfection with *T. muris*, inflammatory Mφs accumulate in the large intestine and become more prevalent than resident Mφs. Importantly, we show for the first time, to our knowledge, that both inflammatory and resident Mφs become alternatively activated following infection with a gastrointestinal nematode. Furthermore, both inflammatory and resident Mφs remain alternatively activated for several weeks after the immunopathology appears to have subsided. That inflammatory Mφs can be alternatively activated reveals an interesting and far-reaching paradox because inflammatory Mφs are thought to amplify inflammation, whereas M2s are implicated in the resolution of inflammation (7, 14, 15, 17, 39).

The proliferation of resident Mφs drives the accumulation of Mφs in the pleural and peritoneal cavities following infection with filarial nematode parasites (18, 19), and the replenishment of Mφs in atherosclerotic lesions depends predominantly on local Mφ proliferation (40). Therefore, in these models of inflammation, Mφ proliferation, rather than monocyte influx, is the principle mechanism underlying the accumulation of Mφs. Although the accumulation of Mφs during the development of colitis has been shown to involve the recruitment of Ly6C^high^CX3CR1^low^ inflammatory monocytes (7, 15, 17), the potential of local resident Mφ proliferation to contribute toward this process has not been investigated previously. During *T. muris* infection, the accumulation of Mφs in the large intestine does not coincide with an increase in the proliferation of resident Mφs. Furthermore, the inhibition of monocyte recruitment greatly impedes the accumulation of Mφs in the gut. Therefore, monocyte recruitment is the principle mechanism of Mφ accumulation during the development of the inflammatory response to *T. muris*. Nevertheless, following worm expulsion, there is a small but significant increase in the proliferation of inflammatory Mφs. Given that, in the large intestine, resident Mφs are derived from inflammatory Mφs (14), the increase in the proliferation of inflammatory Mφs could boost resident Mφ numbers in the late stages of inflammation following worm expulsion.

Importantly, we reveal that the vast majority of M2s do not proliferate in the large intestine at any stage either before or postinfection with *T. muris*. Furthermore, we show that the accumulation of M2s in the large intestine is greatly reduced by disrupting monocyte recruitment to the gut. This is consistent with previous work showing a CCR2-dependent mechanism underlying the recruitment of Ly6C^hi^CCR2^hi^CX3CR1^lo^ blood monocytes to the colon during an inflammatory response (14, 15, 17). Therefore, in contrast to the profound ability of M2s to proliferate in the peritoneal and pleural cavities following infection with filarial nematodes (18, 19), in our model of intestinal inflammation, the accumulation of M2s is largely independent of their self-replication. Instead, M2s are derived predominantly from blood monocytes that migrate to the gut postinfection. This mirrors the recent work by Girgis et al. (41) on the accumulation of M2s in the liver following infection with the trematode parasite *Schistosoma mansoni*. Taken together, it is becoming clear that the mechanisms that underlie the accumulation of M2s following infection with parasitic nematodes are either tissue specific or parasite species specific.

In summary, this study reveals the dynamic changes that take place to the phenotype of Mφ subsets during the initiation, amplification, and resolution of intestinal inflammation. We describe the emergence of M1s during worm infection and M2s following worm expulsion. However, in contrast to previous studies (18, 19), in the large intestine, the accumulation of M2s is chiefly dependent on the recruitment of blood monocytes rather than their proliferation. Understanding the mechanisms that control M1/M2 balance will bring the pharmacological manipulation of Mφs a step closer. The promotion of anti-inflammatory and the restraint of proinflammatory subsets of Mφs have exciting potential for the treatment of a range of debilitating inflammatory diseases.

## Supplementary Material

Data Supplement
