# Supplementary material for: Dynamic Changes in Macrophage Activation and Proliferation during the Development and Resolution of Intestinal Inflammation
Source: J Immunol. 2014 Sep 26;193(9):4684–95. doi: 10.4049/jimmunol.1400502 (PMC4201944; doi:10.4049/jimmunol.1400502)
Supplement: Data Supplement [file supp_193_9_4684__index.html]

Dynamic Changes in Macrophage Activation and Proliferation during the Development and Resolution of Intestinal Inflammation — Data Supplement 

# Dynamic Changes in Macrophage Activation and Proliferation during the Development and Resolution of Intestinal Inflammation

## Data Supplement

**Files in this Data Supplement:**

- Supplemental Figures 1 (PDF)
