# Supplementary material for: Dynamic Changes in Macrophage Activation and Proliferation during the Development and Resolution of Intestinal Inflammation
Source: J Immunol. 2014 Sep 26;193(9):4684–95. doi: 10.4049/jimmunol.1400502 (PMC4201944; doi:10.4049/jimmunol.1400502)
Supplement: Data Supplement [file 1400502_JI_1400502_Supplemental_Figures_1.pdf]

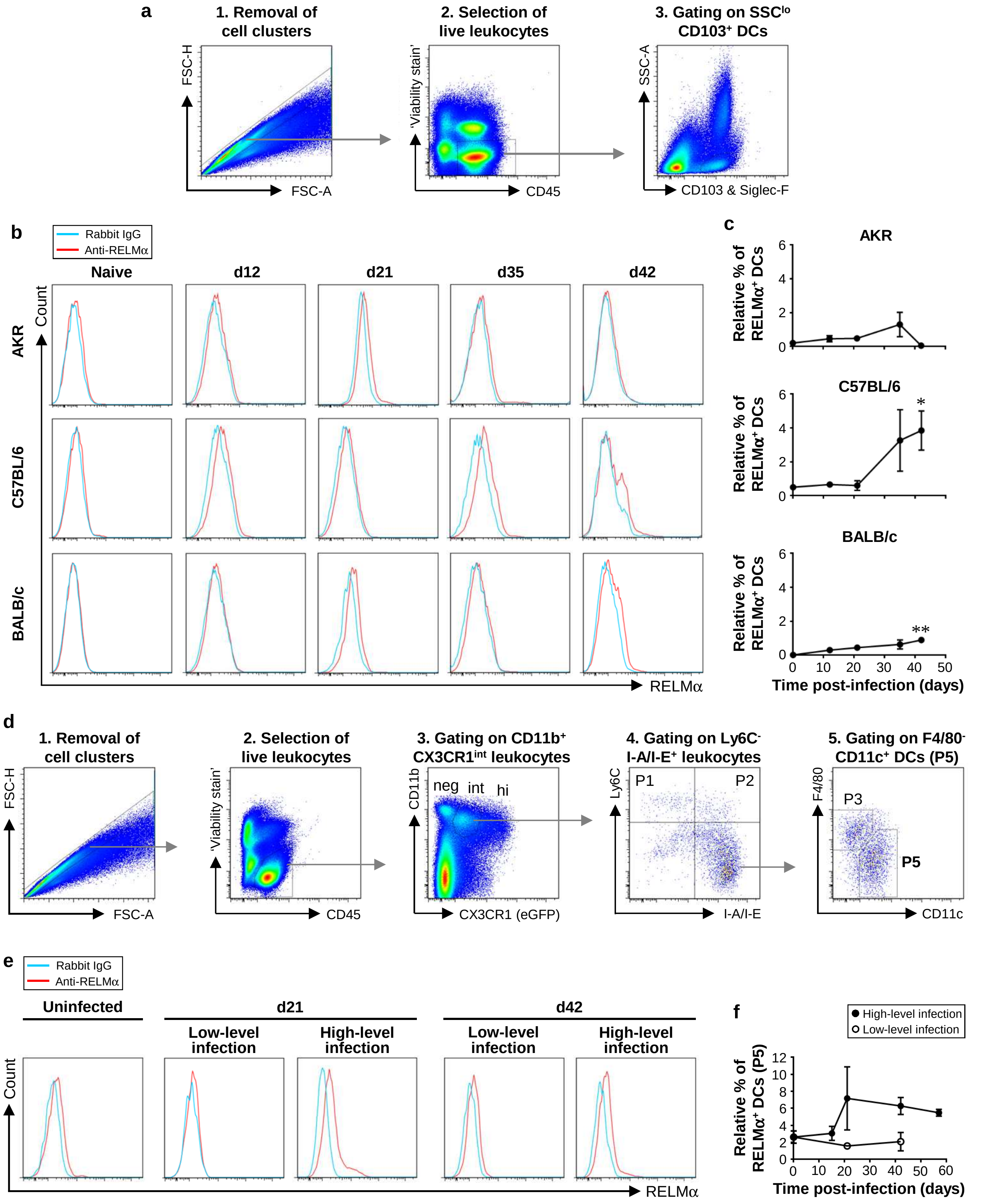

Supplementary Figure 1. A minor fraction of DCs expresses RELMα and there is a small but significant increase in this population in the large intestine post-infection with *T. muris*. Three different strains of mouse (AKR, C57BL/6 and BALB/c) were either left uninfected or infected with a high-level of *T. muris* ova. Cells were isolated from the lamina propria of the caecum and proximal colon, stained with a panel of fluorochrome-labelled antibodies and then analysed by flow cytometry. Live DCs were analysed by gating on 'viability stain'-negative $CD45^{+}CD103^{+}SSC^{lo}$ cells as shown in **a**. Representative histogram plots of RELMα staining are shown in **b**. Quantitative analysis of the staining is shown in **c**. $CX3CR1^{gfp/+}$ mice were infected with either a low-level or a high-level of *T. muris* ova. Another group of $CX3CR1^{gfp/+}$ mice was left uninfected. Cells were isolated from the lamina propria of the caecum and proximal colon, stained with a panel of fluorochrome-labelled antibodies and then analysed by flow cytometry. A population of $CD11b^{+}CX3CR1^{int}Ly6C^{-}I\text{-}A/I\text{-}E^{+}F4/80^{-}CD11c^{+}$ DCs was identified (P5) as shown in **d**. Representative histogram plots of RELMα staining by this subpopulation of DCs (P5) are shown in **e**. Quantitative analysis of the staining is shown in **f**. The values are the means ± SEM of five mice in each group and the results are representative of two separate experiments. *P<0.05 (time-points post-infection compared with uninfected).

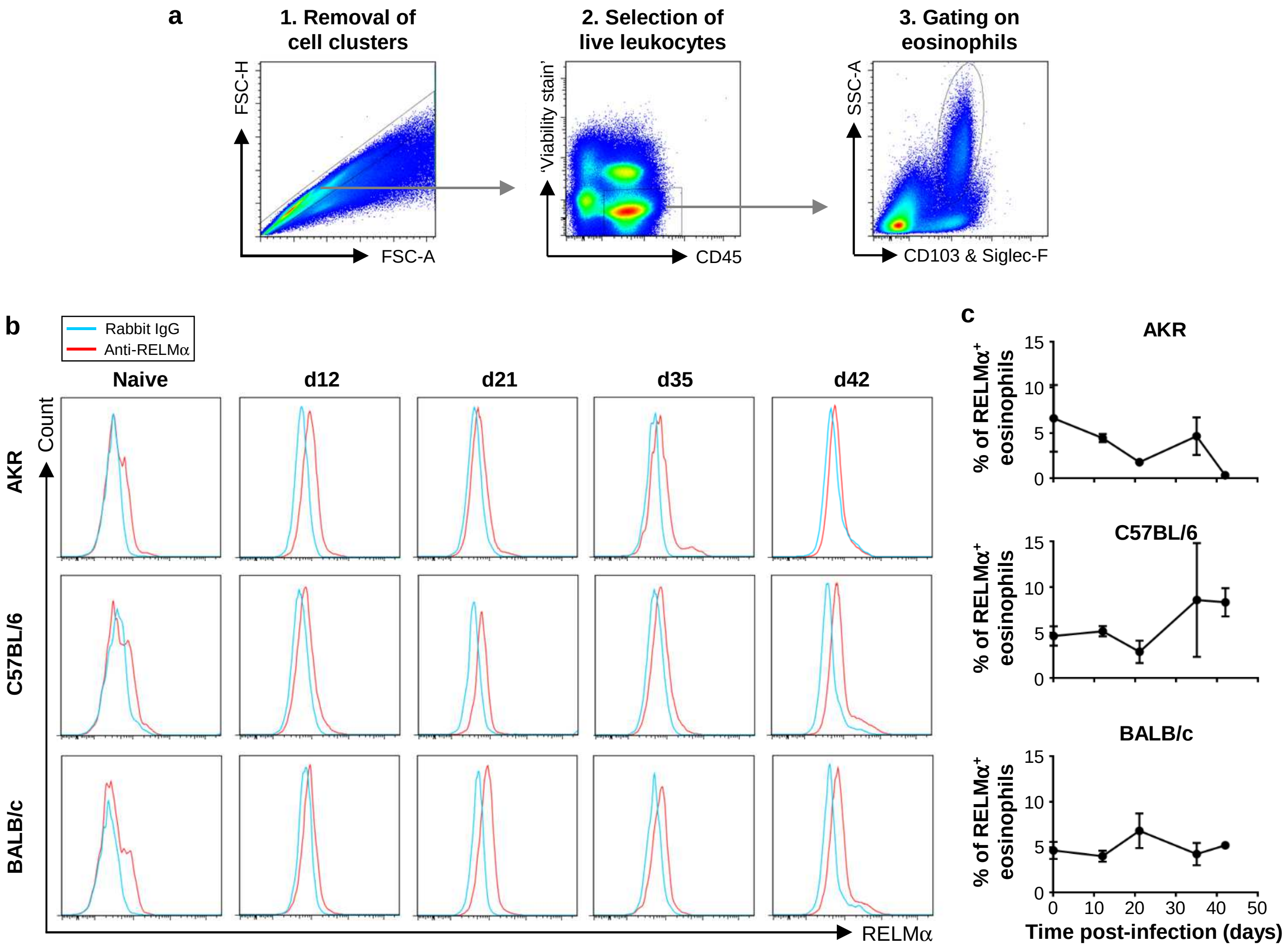

Supplementary Figure 2. A minor fraction of eosinophils expresses RELMα but there is no significant increase in this population in the large intestine post-infection with *T. muris*. Three different strains of mouse (AKR, C57BL/6 and BALB/c) were either left uninfected or infected with a high-level of *T. muris* ova. Cells were isolated from the lamina propria of the caecum and proximal colon, stained with a panel of fluorochrome-labelled antibodies and then analysed by flow cytometry. Live eosinophils were analysed by gating on 'viability stain'-negative $CD45^+$Siglec-$F^+$$SSC^{hi}$ cells as shown in **a**. Representative histogram plots of RELMα staining are shown in **b**. Quantitative analysis of the staining is shown in **c**. The values are the means ± SEM of five mice in each group and the results are representative of two separate experiments.

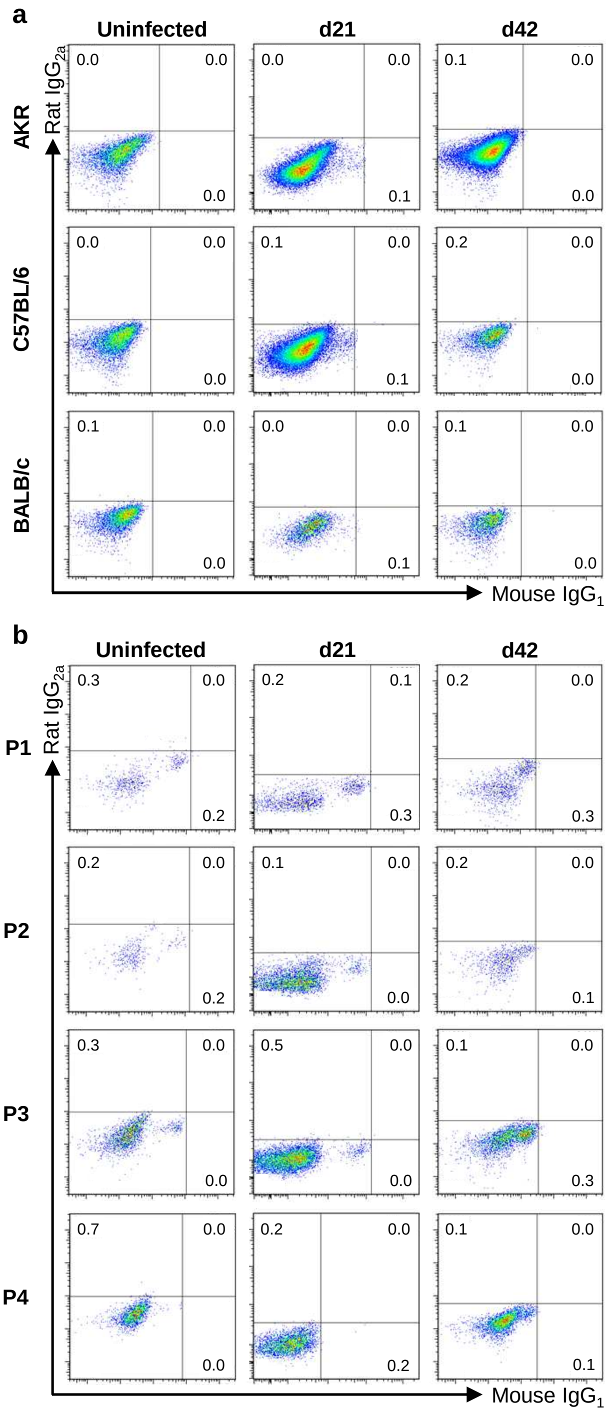

Supplementary Figure 3. Fluorochrome-labelled isotype control antibody staining of macrophages in order to define the gates in Figure 6. AKR, C57BL/6, BALB/c and CX3CR1$^{gfp/+}$ mice were infected with a high-level of *T. muris* ova. Each mouse was injected with 1.5 mg BrdU four hours before it was killed. Cells were isolated from the lamina propria of the caecum and proximal colon, stained with a panel of fluorochrome-labelled antibodies and then analysed by flow cytometry. In AKR, C57BL/6 and BALB/c mice (**a**), live Mφs were analysed by gating on 'viability stain'-negative CD45$^+$CD11b$^+$F4/80$^+$CD103$^-$Siglec-F$^-$ cells (as shown in Fig. 4a). In CX3CR1$^{gfp/+}$ mice (**b**), four populations of monocytes and Mϕs (P1-P4, as defined in Fig. 5a) were analysed. Representative plots of Rat IgG$_{2a}$ (isotype control for the Ki-67 antibody) and Rat IgG$_1$ (isotype control for BrdU antibody) staining at selected time-points post-infection are shown.
